# Beyond privacy calculus: public acceptance of non-invasive therapeutic brain-computer interfaces in China

**DOI:** 10.3389/fpubh.2026.1904359

**Published:** 2026-08-31

**Authors:** Haoyu Wang, Mei Yin

**Affiliations:** College of Humanities and Social Sciences, Harbin Medical University, Harbin, China

**Keywords:** China, cost desensitization, non-invasive brain-computer interface, technology acceptance, usage intention, value-based adoption model

## Abstract

**Background and objective:**

Non-invasive brain–computer interfaces (BCIs) are rapidly transitioning from laboratory settings to clinical rehabilitation and mental health applications. However, unlike ordinary consumer technologies, therapeutic BCIs are adopted under health imperatives rather than discretionary choice. In such contexts, the “privacy calculus” logic underlying the Value-based Adoption Model (VAM), which assumes users rationally weigh benefits against sacrifices, may not adequately capture adoption decisions. This study aimed to explore the public’s acceptance of non-invasive therapeutic BCIs in China, identify influencing factors, and examine the moderating roles of age, monthly income, and disease status.

**Methods:**

Using a cross-sectional survey design, 337 valid questionnaires were collected from Chinese adults via snowball sampling on the Wenjuanxing online platform. A structural equation model (SEM) incorporating perceived benefit, perceived sacrifice, perceived value, and a second-order latent variable “external drivers” was constructed and tested. Hierarchical regression analysis examined moderation effects.

**Results:**

The mean usage intention score was 3.93 (SD = 0.63). Perceived benefit strongly and positively predicted perceived value (*β* = 0.951, *p* < 0.001), while perceived value (*β* = 0.749, *p* < 0.001) and external drivers (*β* = 0.241, *p* = 0.027) positively predicted usage intention. The negative effect of perceived sacrifice on perceived value was non-significant (*β* = −0.091, *p* = 0.061); a post-hoc power analysis confirmed the study was adequately powered (power > 0.99 for a medium effect), suggesting this effect is negligible. Age positively moderated the “perceived sacrifice → perceived value” path (*β* = 0.118, *p* < 0.01), with older respondents showing greater tolerance for technological costs.

**Conclusion:**

In the context of non-invasive therapeutic BCIs, the negative effect of perceived sacrifice on perceived value was not supported, suggesting that the classic privacy calculus logic may be attenuated when health imperatives are salient. We propose “cost desensitization” as a tentative mechanism warranting further mixed-methods validation research. These findings inform clinical expectation management and ethical governance of emerging neurotechnologies.

## 1 Introduction

Non-invasive therapeutic brain-computer interfaces (BCIs) refer to a class of technologies that acquire brain signals via external sensors worn on the scalp without penetrating the skin or skull and use these signals to assist in the treatment of specific brain or neurological disorders (1). In the present study, the term specifically denotes non-invasive devices used by the public in medical settings that support stroke rehabilitation, depression regulation, and sleep disorder intervention through mechanisms such as neurofeedback training and motor imagery decoding (2). Propelled by the national “Brain Science and Brain-Inspired Research” strategic agenda, non-invasive BCIs are rapidly moving beyond the laboratory into clinical rehabilitation and mental health care; however, public awareness and use of this technology in China remain at an early, exploratory stage (3).

Existing research has been dominated by engineering-oriented algorithm optimization or philosophical ethical reflection, leaving the actual acceptance psychology of the public, the ultimate adopters of the technology, largely uninvestigated through quantitative means. More notably, mainstream technology-acceptance research has predominantly relied on assumptions imported from Western consumer contexts, presupposing that users are rational decision-makers who consciously weigh benefits against privacy risks through a “privacy calculus” before adopting a technology (4). Yet the context of therapeutic BCI use is fundamentally distinct from ordinary consumer choice: it constitutes an essential health need bound up with treatment and survival. When survival and recovery become overriding priorities, does the user still rationally weigh the costs of technology adoption, or does sensitivity to such costs become systematically attenuated—a phenomenon we term “cost desensitization”? This question has not yet been empirically examined, yet it bears directly on the design of ethical governance pathways.

Building on this gap, the present study adopts the Value-based Adoption Model (VAM) as its theoretical framework and employs a questionnaire survey to examine the Chinese public’s acceptance intention toward non-invasive therapeutic BCIs and its underlying mechanisms. The study aims to make three contributions. First, it empirically tests, within a high-stakes medical context, whether the classic VAM assumption that perceived sacrifice negatively predicts perceived value—the core premise of privacy calculus—still holds, thereby identifying and delineating the phenomenon of “cost desensitization” and extending the theoretical boundaries of VAM. Second, it introduces age, monthly income, and disease status as moderating variables to reveal heterogeneity across population subgroups along the sacrifice–value pathway, addressing questions of equity in technology adoption. Third, from the perspective of localizing and refining a Western-derived model, it provides empirical evidence to inform the clinical translation, expectation management, and ethical governance of emerging therapeutic neurotechnologies.

## 2 Theoretical background and hypotheses

### 2.1 The Value-based Adoption Model in medical contexts

The VAM (4) conceptualizes users as value maximizers who weigh perceived benefits against perceived sacrifices before forming adoption intentions. Perceived benefit captures treatment efficacy and quality-of-life improvement; perceived sacrifice encompasses time, cognitive effort, privacy risk, and potential side-effect uncertainty (5). In non-invasive BCI contexts, perceived benefit primarily reflects expectations of disease management and functional recovery. Multiple studies confirm that perceived benefit positively influences perceived value and usage intention (H1: perceived benefit → perceived value, positive; H3: perceived value → usage intention, positive).

Classical VAM predicts that perceived sacrifice negatively affects perceived value (H2). However, in healthcare contexts, “survival rationality,” the prioritization of health restoration above all else, may neutralize this negative effect. When patients face life-threatening or quality-of-life-limiting conditions, they may cognitively rationalize technological costs as necessary treatment investments rather than avoidable burdens, yielding what we term “cost desensitization” (6). This study therefore tests H2 with the expectation that it may be rejected in the current context.

### 2.2 External drivers

This study integrates social influence, facilitating conditions, and perceived interest into a second-order latent variable termed “external drivers.” Social influence and facilitating conditions are two core environmental determinants in the Unified Theory of Acceptance and Use of Technology (UTAUT/UTAUT2.) (7, 8). Social influence refers to the normative pressure an individual perceives from important others regarding technology use, whereas facilitating conditions refer to the perceived external resources and technical support available (e.g., device accessibility, operational guidance). Perceived interest, in contrast, reflects an individual’s intrinsic motivation toward the technology itself, resonating with the notion of intrinsic motivation in Self-Determination Theory (9). Following the reciprocal determinism of Social Cognitive Theory (10), behavior is jointly shaped by personal cognitive factors and environmental forces. Although differing in origin, these three constructs jointly constitute driving forces that lie outside the rational cost–benefit appraisal of perceived value in the VAM. They are therefore integrated as the second-order latent variable “external drivers.”

### 2.3 Moderating roles of age, income, and disease status

Prior research indicates that age, income, and health status moderate technology adoption pathways (11), though the underlying mechanisms likely differ. Under the Selection, Optimization, and Compensation (SOC) model, older individuals prioritize maintaining functional health above other goals and therefore allocate cognitive resources toward compensating for technology-related burdens, making them more tolerant of perceived sacrifice (12). Higher income, by contrast, expands users’ financial slack, so monetary and effort-related costs should weigh less heavily in their overall value judgment. Disease status is expected to operate through the cost-desensitization mechanism proposed in Section 2.1, whereby individuals carrying a BCI-relevant diagnosis face a more immediate health imperative than healthy respondents and should therefore show reduced sensitivity to perceived sacrifice. We accordingly introduce age, monthly income, and disease status as moderators of the “perceived sacrifice → perceived value” and “perceived value → usage intention” pathways.

## 3 Materials and methods

### 3.1 Study design and participants

A cross-sectional online survey was conducted between April and August 2025. The target population was Chinese adults (age ≥ 18) with basic Chinese literacy. Recruitment used snowball sampling via the Wenjuanxing online platform, disseminated through social media networks and health-related online communities. Informed electronic consent was obtained from all participants; those who declined or failed attention checks were excluded. The study adhered to the Declaration of Helsinki and all data were anonymized. Of 376 questionnaires collected, 39 were excluded for rule violations (same-option responding, failed attention checks, or implausible response times), yielding 337 valid responses (valid response rate: 89.63%).

### 3.2 Questionnaire design and measures

The questionnaire comprised two modules. Part one covered demographic and health background information (sex, age, education, occupation, monthly income, residential region, self-reported disease status, and prior BCI-related experience); and part two consisted of a 27-item scale covering seven latent constructs. All items used a five-point Likert scale (1 = strongly disagree, 5 = strongly agree).

To ensure a shared understanding of the target technology before responding, the first page of the questionnaire provided a standardized definition of non-invasive therapeutic BCIs: “a class of technologies that, without surgical implantation, read brain signals through head-worn devices (e.g., an EEG cap) and use these signals to help treat specific brain or neurological conditions (e.g., stroke rehabilitation, depression regulation).” A link to an accessible explanatory article was also provided for respondents unfamiliar with the technology. The introductory page further stated that there were no right or wrong answers, that responses were anonymous and used solely for academic research, and that respondents could withdraw at any point if they felt uncomfortable, thereby supporting informed consent and response quality.

Constructs and their sources were as follows. Perceived benefit, perceived sacrifice, and perceived value were adapted from Kim et al. (4), with perceived sacrifice covering both technical complexity and privacy/safety risk. Perceived interest, social influence, and facilitating conditions were adapted from the UTAUT instrument of Venkatesh et al. (7). Usage intention was adapted from Davis (13). All items were reworded to refer specifically to non-invasive therapeutic BCIs. Variable definitions are provided in Table 1.

**Table 1:** Variable definitions.

Variable	Definition
Perceived benefit	User’s overall evaluation of the therapeutic effects and health improvements expected from non-invasive therapeutic BCI
Perceived sacrifice	User’s perceived time, effort, privacy, and risk costs associated with using non-invasive therapeutic BCI
Perceived value	User’s integrated evaluation of benefits versus sacrifices associated with non-invasive therapeutic BCI
Perceived interest	User’s curiosity and exploratory tendency toward non-invasive therapeutic BCI
Social influence	Degree to which the user perceives that important others (e.g., physicians, family) believe they should use the technology
Facilitating conditions	Degree to which the user perceives that organizational and technical conditions can support technology use
Usage intention	User’s subjective likelihood of using non-invasive therapeutic BCI when needed

Content validity was established through blind review by five experts in medicine, ethics, BCI research, and psychometrics. A pilot study (*n* = 60) informed item refinement through item analysis and exploratory factor analysis; items with low loadings or severe cross-loadings were deleted. Exploratory factor analysis further revealed that perceived usefulness and perceived effectiveness loaded on a single factor, as did technical complexity and perceived risk; these pairs were accordingly merged into perceived benefit and perceived sacrifice, respectively, yielding seven latent constructs for confirmatory analysis.

### 3.3 Statistical analyses

All analyses were conducted in R 4.4.0. (i) Descriptive statistics: means, standard deviations, skewness, and kurtosis for all latent variables. (ii) Reliability and validity: Cronbach’s *α* and composite reliability (CR) for internal consistency; confirmatory factor analysis (CFA) for convergent validity (AVE); discriminant validity assessed by comparing the square root of AVE against inter-construct correlations. (iii) Common method bias: Harman’s single-factor test (threshold: <40% variance explained by the first factor). (iv) Structural equation modeling: full SEM with the second-order “external drivers” latent variable; model fit assessed by *χ*^2^/df (<3), CFI and TLI (>0.90), RMSEA (<0.08), SRMR (<0.08). (v) Moderation analysis: hierarchical multiple regression was employed to examine the moderating effects of age, monthly income, and disease status on the “perceived sacrifice → perceived value” and “perceived value → usage intention” pathways. To reduce multicollinearity between main effects and interaction terms, all continuous predictor and moderator variables (perceived benefit, perceived sacrifice, age, monthly income) were mean-centered prior to model estimation by subtracting the sample mean from each observed value; the categorical moderator, disease status, was dummy-coded (0 = healthy, 1 = diagnosed). Interaction terms were constructed by multiplying the centered predictor by the corresponding centered (or dummy-coded) moderator. The hierarchical regression proceeded in two steps: Step 1 (Models 1, 3, 5) entered the main effects of the predictor and moderator to assess their direct association with perceived value, and Step 2 (Models 2, 4, 6) added the corresponding interaction term while controlling for the main effects. A moderation effect was considered established when the interaction term’s regression coefficient was statistically significant (*p* < 0.05) and the change in explained variance (Δ*R*^2^) was significant. For significant moderation effects, simple slope analysis was further conducted, computing and plotting regression slopes at the moderator’s mean ± 1 SD to clarify the direction and pattern of the moderating effect. (vi) Statistical power: a post-hoc power analysis was conducted for the “perceived sacrifice → perceived value” path (*α* = 0.05, *N* = 337) to assess whether the study was adequately powered to detect a meaningful effect.

## 4 Results

### 4.1 Sample characteristics

Sample demographics are presented in Table 2. The sample was approximately balanced by sex (male: 47.2%; female: 52.8%). The majority of respondents were aged 26–40 years (63.5%) with undergraduate (54.0%) or postgraduate (24.3%) education. Monthly income was concentrated in the CNY 5,001–12,000 range (51.6%). Approximately 40.4% of respondents self-reported a BCI-relevant diagnosis (stroke sequelae, depressive disorders, or sleep disorders); 14.8% reported prior experience with related technologies.

**Table 2:** Sample demographic characteristics (*N* = 337).

Variable	Category	*N*	%
Sex	Male	159	47.2
	Female	178	52.8
Age (years)	18–25	53	15.7
	26–30	86	25.5
	31–40	128	38.0
	41–50	48	14.3
	51–60	21	6.2
	≥61	1	0.3
Education	≤Junior high school	5	1.5
	Senior high/vocational	13	3.9
	Associate degree	55	16.3
	Bachelor’s degree	182	54.0
	Postgraduate	82	24.3
Monthly income (CNY)	<3,000	54	16.0
	3,000–5,000	45	13.4
	5,001–8,000	93	27.6
	8,001–12,000	81	24.0
	12,001–20,000	48	14.3
	≥20,001	16	4.7
Disease status	Has BCI-relevant diagnosis	136	40.4
	Healthy/no diagnosis	201	59.6
Prior BCI experience	Yes	50	14.8
	No	258	76.6
	Uncertain	29	8.6

### 4.2 Common method bias

Because all constructs were measured via self-report within a single questionnaire administered on one occasion, common method bias (CMB) was a relevant concern and was addressed through both procedural and statistical safeguards. Procedurally, the introductory page assured respondents that there were no right or wrong answers, that responses were fully anonymous and used solely for academic research, and that participation could be withdrawn at any time without consequence; these design features are recommended remedies for reducing evaluation apprehension and social desirability bias, two of the principal sources of common method variance (14). Statistically, Harman’s single-factor test was conducted by entering all 27 measurement items into an unrotated exploratory factor analysis. The unrotated solution yielded seven factors with eigenvalues greater than 1, and the first (largest) factor accounted for 31.04% of the total variance, well below the conventional 40% threshold and far from explaining the majority of covariance among items. Common method bias is therefore unlikely to substantially distort the findings. Harman’s test is nonetheless a relatively coarse diagnostic that can detect but not fully rule out common method variance; this limitation is acknowledged in Section 5.6.

### 4.3 Descriptive statistics

Descriptive statistics for all constructs are shown in Table 3. Usage intention had a mean of 3.93 (SD = 0.63), reflecting moderate-to-high acceptance. Facilitating conditions (M = 4.02) and social influence (M = 4.00) scored highest; perceived sacrifice scored lowest (M = 3.50). All variables showed skewness between −1.21 and −0.54 and kurtosis between 0.58 and 3.33, consistent with approximate normality.

**Table 3:** Descriptive statistics of core latent variables (*N* = 337).

Variable	Mean	SD	Median	Skew	Kurt
Perceived interest	3.97	0.65	4.00	−0.91	1.17
Perceived benefit	3.84	0.59	3.83	−0.54	0.58
Perceived sacrifice	3.50	0.77	3.50	−0.86	1.11
Perceived value	3.76	0.70	3.67	−0.88	1.30
Social influence	4.00	0.63	4.00	−1.21	3.33
Facilitating conditions	4.02	0.63	4.00	−1.07	2.52
Usage intention	3.93	0.63	4.00	−1.01	1.96

### 4.4 Measurement model

CFA results are summarized in Table 4. Standardized factor loadings ranged from 0.495 to 0.752, all significant at *p* < 0.001. CR ranged from 0.662 to 0.813; Cronbach’s *α* from 0.660 to 0.810, meeting the threshold of ≥ 0.60. AVE values ranged from 0.399 to 0.435, slightly below the ideal threshold of 0.50, but CR values all exceeded 0.60, supporting acceptable convergent validity. For discriminant validity, the square root of AVE exceeded inter-construct correlations for most construct pairs. Several correlations nonetheless exceeded the corresponding AVE square roots by a small margin—perceived benefit with perceived value (0.703), with usage intention (0.708) and with facilitating conditions (0.651), and social influence with facilitating conditions (0.641), so the Fornell–Larcker criterion was not satisfied for every pair. Because the seven-factor measurement model nevertheless fitted substantially better than a single-factor alternative in which all items loaded on one common construct, discriminant validity was judged acceptable overall, subject to the reservations noted in Section 5.6 (Table 5). To further address concerns about the high perceived benefit → perceived value association, we conducted a competing-model comparison and multicollinearity diagnostics. This targeted comparison, which merges only these two constructs rather than collapsing all seven, showed that the merged model did not fit significantly worse (Δ*χ*^2^ = 11.11, Δdf = 6, *p* = 0.085); at the same time, all variance inflation factors were below 5 (maximum = 2.17), indicating that multicollinearity did not distort parameter estimates. The high correlation is consistent with VAM theory, in which perceived benefit is the principal antecedent of perceived value (4).

**Table 4:** Reliability and validity results.

Construct	Items	CR	AVE	*α*	Factor loadings
Perceived interest	A1–A3	0.662	0.399	0.660	0.495–0.703
Perceived benefit	B1–B3, C1–C3	0.813	0.421	0.810	0.609–0.698
Perceived sacrifice	D1, D3, E1, E2	0.750	0.435	0.746	0.519–0.752
Perceived value	F1–F3	0.688	0.425	0.706	0.613–0.706
Social influence	G1–G3	0.675	0.410	0.669	0.603–0.665
Facilitating conditions	H1–H3	0.673	0.411	0.663	0.517–0.701
Usage intention	I1–I3	0.694	0.431	0.696	0.639–0.695

CR, composite reliability; AVE, average variance extracted; *α*, Cronbach’s alpha. All standardized loadings significant at *p* < 0.001.

**Table 5:** Discriminant validity and inter-construct correlation matrix.

	1 PI	2 PB	3 PS	4 PV	5 SI	6 FC	7 UI
1. Perceived interest	**0.632**						
2. Perceived benefit	0.625^***^	**0.649**					
3. Perceived sacrifice	0.005	−0.049	**0.660**				
4. Perceived value	0.514^***^	0.703^***^	−0.098	**0.652**			
5. Social influence	0.611^***^	0.603^***^	0.009	0.528^***^	**0.640**		
6. Facilitating conditions	0.575^***^	0.651^***^	0.033	0.510^***^	0.641^***^	**0.641**	
7. Usage intention	0.565^***^	0.708^***^	−0.091	0.626^***^	0.572^***^	0.535^***^	**0.656**

Diagonal entries (bold) are the square roots of AVE. ^***^p < 0.001. PI, Perceived Interest; PB, Perceived Benefit; PS, Perceived Sacrifice; PV, Perceived Value; SI, Social Influence; FC, Facilitating Conditions; UI, Usage Intention. Note the diagonal value 0.641 is distinct from the Social Influence × Facilitating Conditions correlation, also 0.641.

### 4.5 Structural equation model

The three constructs—perceived interest, social influence, and facilitating conditions—showed inter-correlations ranging from 0.575 to 0.641, and integrating them as the second-order latent variable “external drivers” yielded variance inflation factors below 5, preventing multicollinearity while operationalizing the environmental push function. The final SEM demonstrated good fit: *χ*^2^/df = 1.702, CFI = 0.937, TLI = 0.929, RMSEA = 0.046, SRMR = 0.047 (Table 6).

**Table 6:** Structural equation model path coefficients and hypothesis test results.

H	Path	*β*	SE	*p*	95% CI	Result
H1	Perceived benefit → perceived value	0.951^***^	0.025	<0.001	[0.901, 1.000]	Supported
H2	Perceived sacrifice → perceived value	−0.091	0.048	0.061	[−0.186, 0.004]	Not supported
H3	Perceived value → usage intention	0.749^***^	0.103	<0.001	[0.548, 0.950]	Supported
H4	External drivers → usage intention	0.241^*^	0.109	0.027	[0.028, 0.453]	Supported

Model fit: *χ*^2^/df = 1.702; CFI = 0.937; TLI = 0.929; RMSEA = 0.046; SRMR = 0.047. *R*^2^ = 0.927 for perceived value and 0.910 for usage intention (latent-variable estimates; observed-score equivalents are reported in Section 4.5). *β*, SE, and 95% CI are from the fully standardized solution. *p < 0.05; **p < 0.01; ***p < 0.001.

**Figure 1:**
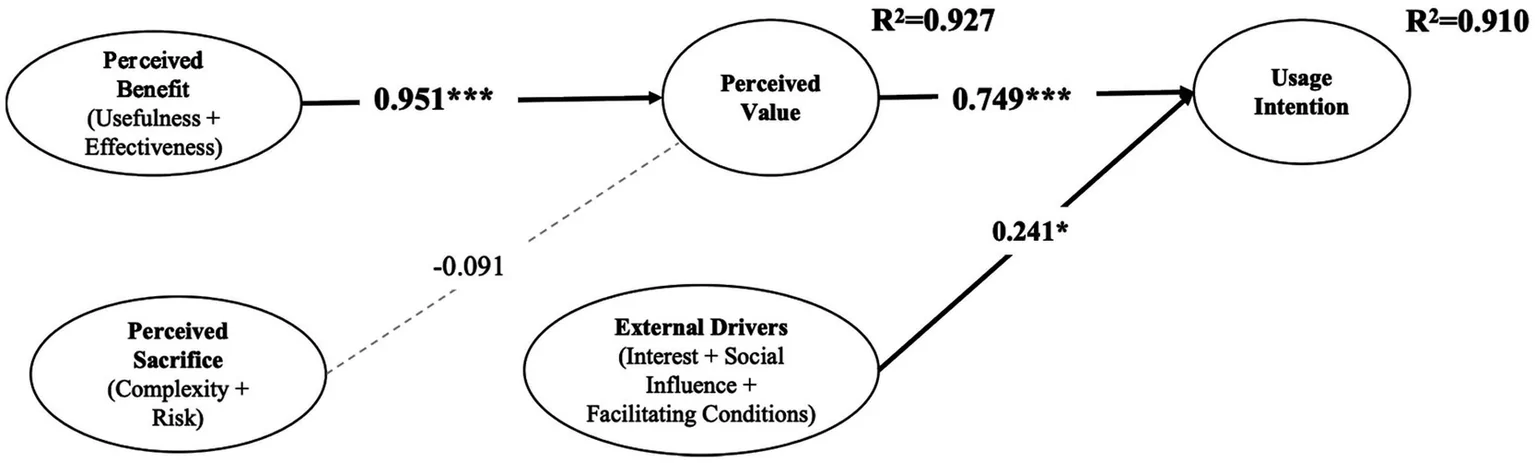
Structural equation model path diagram for public acceptance of non-invasive therapeutic BCIs. Solid arrows indicate significant paths; the dashed arrow indicates the non-significant “perceived sacrifice → perceived value” path. *R*^2^ values are from the latent-variable model and are corrected for measurement error; observed-score equivalents are reported in Section 4.5. *β*, SE, and 95% CI are from the fully standardized solution. ^***^p < 0.001; ^*^p < 0.05.

Path coefficients are presented in Table 6 and Figure 1. Perceived benefit significantly and positively predicted perceived value (*β* = 0.951, *p* < 0.001), supporting H1. The negative effect of perceived sacrifice on perceived value was non-significant (*β* = −0.091, *p* = 0.061), and H2 was not supported. A post-hoc power analysis indicated that, with *N* = 337 and *α* = 0.05, the study had a statistical power of >0.99 to detect a medium effect (*f*^2^ = 0.15) and 0.63 to detect a small effect (*f*^2^ = 0.02) for this path; the minimum detectable effect at 80% power was *f*^2^ = 0.029. This suggests the sample was adequately powered to detect any negative effect of practical significance, indicating that the non-significant result more plausibly reflects a genuinely negligible effect than insufficient power. Nonetheless, power to detect effects smaller than *f*^2^ = 0.02 was limited, so a very weak negative effect cannot be entirely excluded. Perceived value significantly and positively predicted usage intention (*β* = 0.749, *p* < 0.001), supporting H3. External drivers significantly and positively predicted usage intention (*β* = 0.241, *p* = 0.027), supporting H4. The model accounted for a substantial proportion of variance in both endogenous constructs (*R*^2^ = 0.927 for perceived value; *R*^2^ = 0.910 for usage intention). These estimates derive from a latent-variable model in which structural paths are corrected for measurement error, so they represent disattenuated upper bounds rather than values directly comparable with studies that model composite or summated scores. To gauge the magnitude of this difference, we re-estimated the same structural relations using observed composite scores (item means for each construct) in OLS regression. The observed-score model yielded *R*^2^ = 0.498 for perceived value and *R*^2^ = 0.508 for usage intention; the latent estimates are therefore approximately 1.86 and 1.79 times their observed-score counterparts. The observed-score values fall within the 0.40–0.70 range typically reported for behavioral intention in the VAM/UTAUT literature—for reference, the original UTAUT explained approximately 70% of the variance in behavioral intention (7), and UTAUT2 explained 74% with interaction terms included and 44% for direct effects only (8). The discrepancy between the two sets of estimates indicates that the latent *R*^2^ values are inflated both by measurement-error correction, substantial here given the modest AVE and CR values reported in Section 4.4, and by the conceptual proximity of perceived benefit and perceived value discussed in Sections 4.4 and 5.6. The latent estimates should accordingly be read as theoretical upper bounds, with the observed-score values providing the more appropriate benchmark for comparison with published work.

### 4.6 Moderation analysis

Hierarchical regression with usage intention as the dependent variable showed that age (*β* = 0.132, *p* < 0.05) and monthly income (*β* = 0.128, *p* < 0.05) were significant positive predictors. However, the adjusted *R*^2^ of this demographic-only model was only 0.037, indicating that demographic characteristics explain far less variance in usage intention than do cognitive variables (SEM *R*^2^ = 0.910).

**Figure 2:**
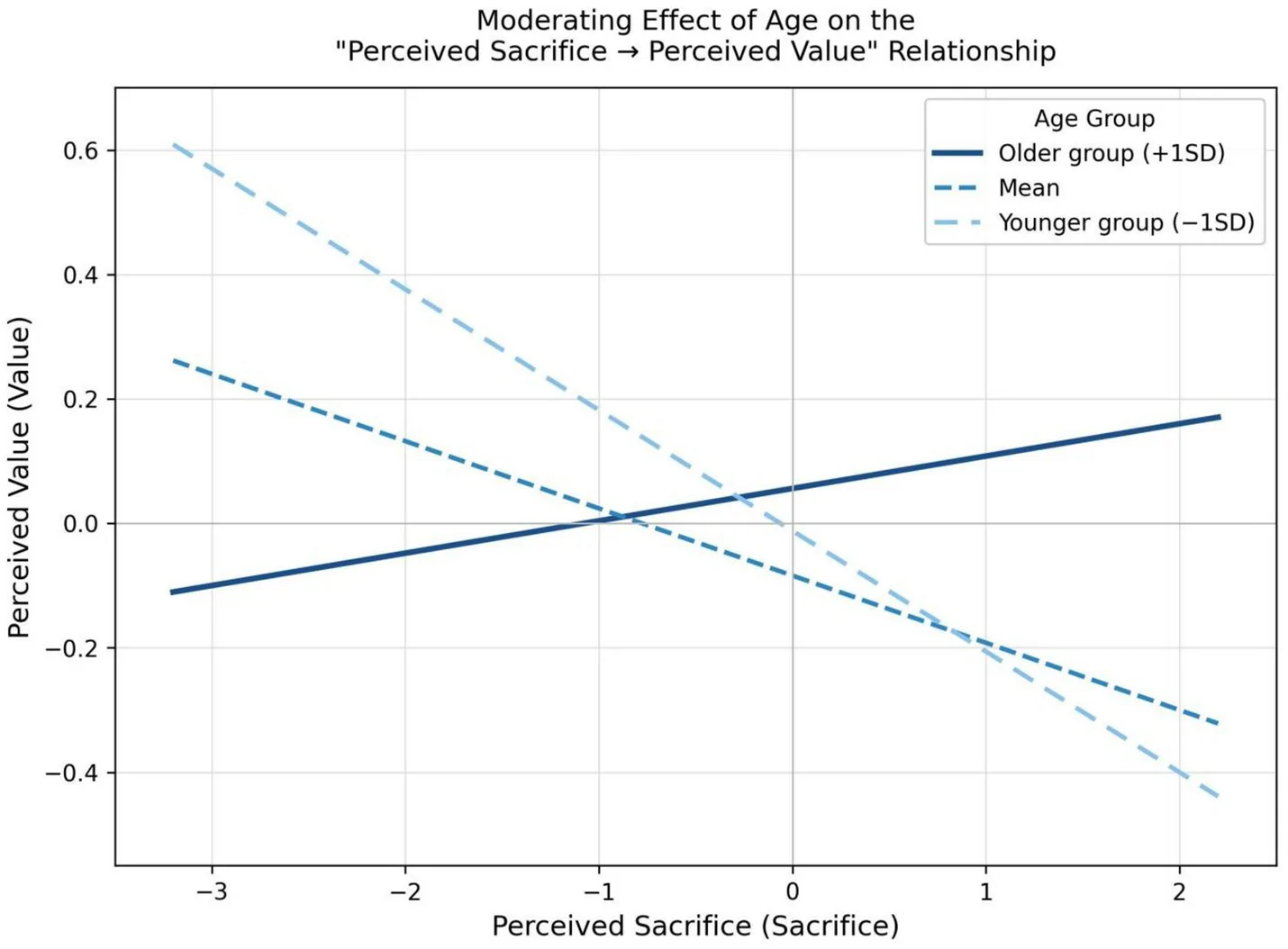
Simple slope analysis of the moderating effect of age on the “perceived sacrifice → perceived value” pathway. The steep negative slope for the low-age group indicates that younger respondents’ perceived value decreases sharply as perceived sacrifice increases; the relatively flat slope for the high-age group indicates substantially greater cost tolerance in older respondents.

In the moderation analysis, only age showed a significant positive moderating effect on the “perceived sacrifice → perceived value” path (*β* = 0.118, *p* < 0.01; Table 7). Simple slope analysis revealed that for younger respondents, perceived value decreased steeply as perceived sacrifice increased, whereas for older respondents the slope was substantially flatter (Figure 2), indicating greater cost tolerance with advancing age. The moderating effects of monthly income and disease status on all tested pathways were non-significant (*p* > 0.05).

**Table 7:** Moderating effect of age on the “perceived sacrifice → perceived value” pathway.

Predictor	*β*	SE	*t*	*p*	95% CI	Notes
Perceived benefit	0.686^***^	0.0386	17.748	<0.001	[0.610, 0.762]	
Perceived sacrifice	−0.070	0.0389	−1.792	0.074	[−0.146, 0.007]	
Age	0.050	0.0389	1.288	0.199	[−0.026, 0.127]	
Age × perceived sacrifice	0.118**	0.0405	2.909	0.004	[0.038, 0.197]	Significant moderation

*R*^2^ = 0.514; *F* = 87.698^***^. Continuous variables were mean-centered. *β*, SE, and 95% CI are from the fully standardized solution. ^*^p < 0.05; ^**^p < 0.01; ^***^p < 0.001.

## 5 Discussion

### 5.1 Cost desensitization: a boundary condition for the VAM

A notable finding is the non-significant effect of perceived sacrifice on perceived value (*β* = −0.091, *p* = 0.061). Supported by the power analysis above, this null result is unlikely to stem from insufficient sample size. Instead of concluding that privacy calculus is absent, we interpret this as evidence that its negative effect may be substantially attenuated in Chinese medical contexts where health imperatives dominate. We tentatively propose “cost desensitization” as a mechanism that requires further confirmatory testing, whereby patients appear to rationalize neural privacy risks and operational burdens as necessary therapeutic costs. This parallels the privacy–personalization paradox documented in mobile health research (15), just as users voluntarily disclose sensitive data in exchange for personalized healthcare, patients facing urgent health imperatives accept perceived sacrifices that would otherwise deter adoption. The non-invasive nature of the technology may further attenuate risk perception by providing a psychological “safe harbor,” absent physical intrusion, potential harm feels intuitively less salient.

This phenomenon can be further grounded in two complementary strands of behavioral decision research. First, prospect theory holds that individuals evaluate outcomes relative to a reference point rather than in absolute terms, and become risk-seeking in the domain of losses, departing systematically from the rational trade-off assumed by expected utility theory (16). This logic has been used to explain seemingly irrational treatment choices among seriously ill patients. As health declines and patients come to frame their situation from a loss reference point, they tend to choose riskier or more burdensome interventions and accept costs they would otherwise avoid. Second, a broader body of patient-preference research corroborates that benefit–risk trade-offs are not fixed but shift systematically with clinical circumstance. A systematic review of 105 studies on patient-based benefit–risk assessment across chronic diseases found that greater disease severity and prior treatment experience, rather than time since diagnosis per se, were the dominant drivers of increased risk tolerance, with patients facing more severe symptoms or disease progression consistently more willing to accept higher risk or burden in exchange for efficacy (17). Read together, these two literatures suggest that our BCI patients’ insensitivity to perceived sacrifice is not an isolated artifact but a specific instance of a well-documented pattern: under sufficiently high health stakes, the reference point against which costs are judged shifts, and the classic rational cost–benefit calculus embedded in the VAM is supplanted by reference-dependent, severity-contingent decision logic.

The strong path from perceived benefit to perceived value (*β* = 0.951, *p* < 0.001) is consistent with VAM theory, in which perceived value is defined as the trade-off centered on benefits, making perceived benefit its principal antecedent (4). Instruments developed specifically for medical technology acceptance in Chinese settings have likewise treated benefit- and value-related perceptions as closely associated dimensions (18). This reinforces our interpretation that health-outcome expectations so dominate the value judgment that sacrifice-related considerations are effectively crowded out. Future research should investigate whether this asymmetric value architecture is specific to life-threatening or quality-limiting conditions, or extends more broadly across Chinese healthcare technology contexts.

### 5.2 Age as a moderator: selection, optimization, and compensation

Age significantly moderated the “perceived sacrifice → perceived value” path (*β* = 0.118, *p* < 0.01), with older adults displaying markedly greater cost tolerance. This finding contradicts stereotypes of older users as technology-averse due to cognitive decline. Instead, it aligns with the Selection, Optimization, and Compensation (SOC) theory (12), in that older individuals, motivated to maintain independent functioning and health as their primary life goal, preferentially allocate cognitive resources toward technologies that compensate for physical decline, effectively minimizing the perceived weight of learning costs. Younger respondents, possessing higher health baselines, treat BCI as an enhancement rather than a necessity and thus apply stricter cost–benefit standards. This SOC-based interpretation should nonetheless be read with caution. Because participants were recruited via snowball sampling through social media, the older respondents captured here may themselves be unusually digitally literate and technology-receptive relative to the broader older population, which could have inflated the observed age moderation effect rather than reflecting a general property of older BCI users (Section 5.6).

### 5.3 External drivers

External drivers significantly predicted usage intention (*β* = 0.241, *p* = 0.027), confirming the UTAUT proposition that a supportive social and institutional environment amplifies adoption willingness (7). Clinician recommendations, peer influence, device accessibility, and technology curiosity collectively constitute an ecological push that shapes the adoption landscape independently of personal cost–benefit calculations. This finding echoes research on older adults’ mobile health adoption (19), and highlights the importance of clinical communication, community support networks, and infrastructure investment in facilitating BCI uptake.

### 5.4 Perceived value as a mediator

Perceived value significantly predicted usage intention (*β* = 0.749, *p* < 0.001), consistent with the core VAM proposition (4). The indirect effect of perceived benefit on usage intention, fully mediated through perceived value, confirms that health-outcome expectations constitute the primary driver of adoption. From a clinical practice perspective, these findings recommend shifting emphasis from generic risk disclosure toward expectation management: helping patients form calibrated and realistic expectations of treatment efficacy may be more effective in supporting informed consent than listing potential risks.

### 5.5 Implications for ethical governance

The cost desensitization phenomenon carries important implications for neuroethical governance. If patients systematically underweight privacy sacrifice when health needs are acute, purely autonomy-based informed consent frameworks may be insufficient to protect neural data and human agency in clinical BCI contexts. Regulatory frameworks should proactively embed privacy-by-design principles, restrict commercial exploitation of neural data, and establish shared governance structures that do not depend solely on patients’ self-protective rationality. Clinicians should incorporate expectation calibration into consent processes to prevent therapeutic misconception, i.e., unrealistic beliefs about cure probability, which could expose vulnerable patients to psychological and financial harm. At the policy level, the significant positive effect of monthly income on usage intention (*β* = 0.128, *p* < 0.05) signals an emerging affordability barrier. Preventing BCI technology from becoming a biological privilege of the wealthy will require tiered insurance coverage, public procurement programs, and rental access schemes.

### 5.6 Limitations

Several limitations warrant consideration. First, AVE values for some constructs fell marginally below the ideal 0.50 threshold, and several inter-construct correlations slightly exceeded the corresponding AVE square roots, indicating imperfect discriminant validity. Future studies should refine item sets. Second, the high path coefficient between perceived benefit and perceived value (*β* = 0.951) suggests possible conceptual overlap between these two constructs, and the same overlap together with the disattenuation inherent in latent-variable estimation inflates the explained variance reported by the model. As shown in Section 4.5, re-estimating the structural relations with observed composite scores reduced R^2^ from 0.927 to 0.498 for perceived value and from 0.910 to 0.508 for usage intention. The latent *R*^2^ values should therefore not be interpreted as evidence of unusually strong predictive power, and warrant the same caution we have applied to the discriminant validity findings above. Future work might address this through experimental manipulations, alternative model specifications, or item sets that more sharply distinguish benefit appraisal from value appraisal. We note, however, that our central finding, the non-significant perceived sacrifice → perceived value path, does not depend on the magnitude of the explained variance; it rests on the coefficient estimate and the post-hoc power analysis reported in Section 4.5. Third, the snowball sampling approach via social media platforms introduced selection bias toward younger, more educated, and digitally literate respondents, with older adults and digitally disadvantaged groups underrepresented; the cross-sectional design further precludes causal inference. As noted in Section 5.2, this bias bears particularly on the age-moderation finding, whose magnitude should therefore be read with caution. Longitudinal and probability-based or offline sampling designs covering diverse age and digital-literacy strata are needed to strengthen external validity and to test the moderating role of age more rigorously. Fourth, because “cost desensitization” is inferred from quantitative data, future mixed-methods studies incorporating interviews or open-ended responses are needed to directly examine whether such reasoning occurs. Finally, common method bias was assessed only via Harman’s single-factor test, which offers a useful but relatively coarse diagnostic; future studies should complement it with more rigorous statistical controls, such as an unmeasured latent method factor or a marker-variable approach.

## 6 Conclusion

This study provides one of the first large-sample empirical investigations of public acceptance of non-invasive therapeutic BCIs in China. Our findings demonstrate that, in healthcare contexts characterized by strong health imperatives, the negative effect of perceived sacrifice on perceived value underlying the classic privacy calculus logic was not supported and may be attenuated: perceived benefit dominates value formation to a striking degree. We advance “cost desensitization” as a tentative explanatory concept to be further validated through mixed-methods research, rather than an established phenomenon. This “cost desensitization” phenomenon expands the theoretical boundary of the VAM and underscores the need for context-sensitive adaptations of Western technology acceptance models in Chinese medical settings. Age significantly moderates cost tolerance, consistent with SOC theory. External drivers including social influence, facilitating conditions, and perceived interest constitute a powerful environmental complement to cognitive value judgments. These findings collectively call for patient-centered expectation management in clinical settings, privacy-by-design product development, and equitable access policies to prevent an emerging “biological divide” in access to frontier neurotechnologies.

## Data Availability

The datasets presented in this article are not readily available because the data that support the findings of this study are available from the corresponding author upon reasonable request. The data are not publicly available due to privacy considerations, as participants were assured of data confidentiality at the time of consent. Requests to access the datasets should be directed to MY; Email: dryinmei@163.com.
